# Transcriptomic changes arising during light-induced sporulation in *Physarum polycephalum*

**DOI:** 10.1186/1471-2164-11-115

**Published:** 2010-02-17

**Authors:** Israel Barrantes, Gernot Glockner, Sonja Meyer, Wolfgang Marwan

**Affiliations:** 1 International Max Planck Research School, Magdeburg, Germany; 2 Leibniz Institute for Age Research, Fritz Lipmann Institute, Jena, Germany; 3 Institute for Biochemistry University of Cologne, Joseph-Stelzmann-Str. 52, Cologne, Germany; 4 Max Planck Institute for Dynamics of Complex Technical Systems and Magdeburg Centre for Systems Biology (MaCS), Otto von Guericke University, Magdeburg, Germany; 5 Berlin Center for Genomics in Biodiversity Research Leibniz Institute for Freshwater Ecology and Inland Fisheries, Müggelseedamm 301, D-12587 Berlin, Germany; 6 Magdeburg Centre for Systems Biology (MaCS), Otto von Guericke University, Sandtorstr. 1, D-39106 Magdeburg, Germany

## Abstract

**Background:**

*Physarum polycephalum *is a free-living amoebozoan protist displaying a complex life cycle, including alternation between single- and multinucleate stages through sporulation, a simple form of cell differentiation. Sporulation in *Physarum *can be experimentally induced by several external factors, and *Physarum *displays many biochemical features typical for metazoan cells, including metazoan-type signaling pathways, which makes this organism a model to study cell cycle, cell differentiation and cellular reprogramming.

**Results:**

In order to identify the genes associated to the light-induced sporulation in *Physarum*, especially those related to signal transduction, we isolated RNA before and after photoinduction from sporulation- competent cells, and used these RNAs to synthesize cDNAs, which were then analyzed using the 454 sequencing technology. We obtained 16,669 cDNAs that were annotated at every computational level. 13,169 transcripts included hit count data, from which 2,772 displayed significant differential expression (upregulated: 1,623; downregulated: 1,149). Transcripts with valid annotations and significant differential expression were later integrated into putative networks using interaction information from orthologs.

**Conclusions:**

Gene ontology analysis suggested that most significantly downregulated genes are linked to DNA repair, cell division, inhibition of cell migration, and calcium release, while highly upregulated genes were involved in cell death, cell polarization, maintenance of integrity, and differentiation. In addition, cell death- associated transcripts were overrepresented between the upregulated transcripts. These changes are associated to a network of actin-binding proteins encoded by genes that are differentially regulated before and after light induction.

## Background

*Physarum polycephalum*, commonly known as "slime mold", belongs to the mycetozoan group of Amoebozoa. *Physarum *follows a complex life cycle with haploid and diploid cell types, formed in temporal order as triggered by environmental stimuli [1]. The plasmodium is a multinucleate single cell whose nuclei display natural synchrony with respect to cell cycle and differentiation status. During several days of starvation, a plasmodium grown to macroscopic size becomes competent to sporulate. Sporulation can then be triggered experimentally by exposing a competent plasmodium to a pulse of blue or far-red light, or to heat shock. As plasmodial nuclei are synchronous, and because the timing of the differentiation program in individual plasmodia can be reproduced experimentally, the stage-specific gene expression program that leads to sporulation can be analyzed at high resolution [2-4]. Recent large scale cDNA surveys [5,6] provide a basis for studying the phenomenon at the transcriptomic level.

In order to identify the differentially expressed genes associated with the commitment to sporulation, we characterized and compared two cDNA libraries prepared from competent and light-induced plasmodia using massive parallel sequencing technology [7]. We employed this method because it does not rely on reference transcripts for quantitation, previous cloning steps are not required, it does not have an upper limit for quantitation, and it is a relatively unbiased approach [8]. From the comparison of annotations and transcript quantitations, we found that most differentially expressed genes encode proteins associated to a network of actin-binding proteins. Components of this putative interaction network are associated to development, DNA repair, cell division, calcium release, cell death, and maintenance of cell integrity.

## Results

### Sequencing and Profiling of cDNAs expressed in competent and light-induced plasmodia

Separate cDNA libraries were constructed from polyA^+ ^RNA isolated from two sources: (*i*) competent plasmodia; and (*ii*) sporulation- induced plasmodia (competent plasmodia harvested six hours after exposure to far-red light). The cDNAs libraries were then analyzed using massive parallel sequencing [7,8]. Transcripts were annotated at every bioinformatic level, and the annotation data was used to infer hypothetical interaction networks from differentially regulated genes. The whole approach is summarized in the [Additional File 1: Figure S1].

From the pyrosequencing, we obtained a total output of 61.9 Mb from two runs, corresponding to the starved (26.1 Mb) and light-induced (35.8 Mb) plasmodia libraries. Considering that *Physarum *possess a 300 Mb genome, and assuming that 10% is encoding genes, we estimate a 2.06× coverage of protein coding sequences. The assembled sequencing output consisted of 26,037 sequences, and large cDNAs from this assembly (>500 bp) were then joined to a previously available sequence dataset [6], to form a comprehensive set of representative transcripts. This analysis produced 16,669 sequences, 13,169 of these containing transcript abundance data: 125,456 reads from competent and 99,632 reads from light-induced plasmodia, respectively [Additional File 2]. We used this abundance data (number of reads for each assembled transcript) as a measure of expression, which we defined as "hit counts". The remaining contigs without hit counts consisted of previously sequenced clones from a normalized cDNA library prepared from competent plasmodia [6], indicating that the normalization produced a broader coverage of transcripts. From 11,399 cDNA contigs detected in the competent plasmodia library (10,689 in light-induced plasmodia), over 4,227 were represented with at least five hits (3,553 in light-induced plasmodia [Additional File 3: Figure S2]). Conversely, 8,711 transcripts (52,3%) were found with 5 or less sequence hits in both samples. For statistical reasons, no statement on the differential expression from this fraction could be made. Between contigs with lowest hit counts, 2,437 cDNA species were represented by just one hit (competent plasmodia), and 2,621 from light-induced sample [Additional File 3: Figure S2].

We compared the transcript hit counts between different libraries as a measure of differential gene expression. As most contig species were represented by low hit counts, we normalized the number of hits. To this end, we first obtained the relative frequency (number of hits divided by the total hits on a given condition), and later we calculated the relative frequencies for each contig in the two cDNA samples compared to each other. Given that each EST belongs to a single gene, the significance of its differential expression depends only on the number of hits, respect to the total number of hits on each library [9]. Following these assumptions, we found 2,772 cDNAs that displayed significant differential expression (*P*-value < 0.05). All contig species, regardless of whether differentially expressed or not were submitted to the Sequence Read Archive subset of GenBank [10] (Accession numbers SRX012830 and SRX012831).

The newly assembled contigs were compared against sequence databases using BLASTX [11]. This analysis revealed that 3,310 sequences have significant similarity (≤ 1 × 10-15) to existing sequences in SwissProt [12], 3,651 to the protozoa subset from RefSeq [13], and 3,345 to proteins of the related model organism *Dictyostelium discoideum *(dictyBase; [14]). From the 13,169 sequences with hit counts data, orthologs were identified for 5,544 transcripts (1,287 of these with significant differential expression; [Additional File 4]).

**Figure 1:**
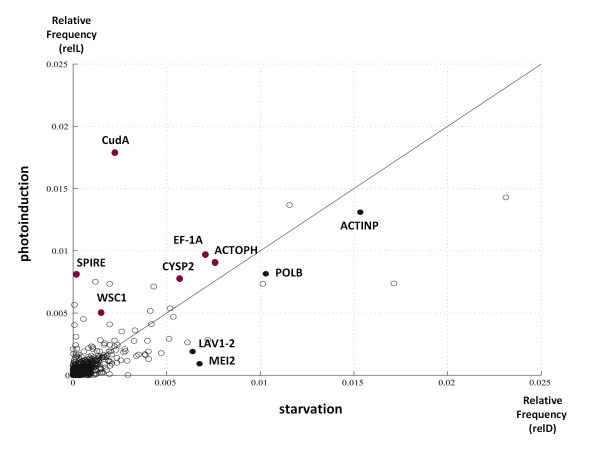
**Relative frequencies of transcripts in libraries prepared from competent and photoinduced plasmodia**. Each circle represents a single cDNA, plotted according to its relative frequencies (number of hits per transcript divided by the total number of hits) on each cDNA library. *relL *and *relD *represent the relative frequencies in the libraries prepared from light-induced and competent plasmodia, respectively. Transcripts more abundant in light-induced (above the diagonal) or in competent, not light-induced plasmodia (below the diagonal) are shown, and SwissProt orthologs are indicated for 10 contigs with relative frequencies greater than 0.005. Red dots represent those contigs which are more abundant in the light-induced plasmodial library, and black those which are more abundant in the library prepared from competent plasmodia. Detailed descriptions for these transcripts can be found on the Table S1.

Later, in order to identify differentially regulated genes, we clustered the contig species into expression groups according to their relative frequencies in both conditions. As a result, we found contigs encoding orthologs related to cell division (meiosis-related protein MEI2; DNA polymerase beta; actin) and protein synthesis and degradation (elongation factor 1- alpha; cathepsin-L cysteine protease) with higher relative frequencies in the competent plasmodial library. Similarly, we found orthologs related to the cytoskeleton (spire; actophorin; cell wall integrity and stress response component, WSC1) and cell differentiation genes (CudA) with greater relative frequencies in the light- induction library (Figure 1; [Additional File 5: Table S1]).

### Gene Ontology Annotation of the Transcriptome

The Gene Ontology (GO) project [15] is an annotation framework that provides a standardized vocabulary that is used to assign function to uncharacterized sequences, based on three main categories: biological processes (BP), molecular functions (MF) and cellular components (CC). We employed BLAST2GO [16], a tool that associates GO terms to sequences based in several annotation evidences, to classify gene function in our dataset. Using the BLASTX hits (annotation e-value cutoff < 1 × 10-6), together with GO terms previously extracted from InterPro domain searches [17], we inferred 13,068 GO annotations for 3,304 (20%) cDNAs, with 11,446 annotations belonging to 2,459 sequences with hit counts data. Transcripts were associated to biological processes (n = 2,437; 15%), molecular functions (n = 2,801; 17%), and cellular components (n = 2,023; 12%). As many as 2,136 (13%), 1,663 (10%) and 1,645 (10%) sequences were annotated with a combination of MF and BP terms, MF and CC, and BP and CC terms respectively, and 1,487 cDNAs were annotated with MF, BP and CC terms altogether. Later, in order to analyze the differences between the two condition groups with respect to the GO annotations, we carried out Fisher exact tests using the Gossip module [18] from BLAST2GO. We found that the 'cell development' (GO:0048468), 'cell death' (GO:0008219) and 'death' (GO:0016265) GO terms were overrepresented in cDNAs with higher relative frequencies in light-induced plasmodia (false discovery rate < 0.01), as compared to competent plasmodia [Additional File 6: Table S2].

### Pathway classification of transcripts

**Figure 2:**
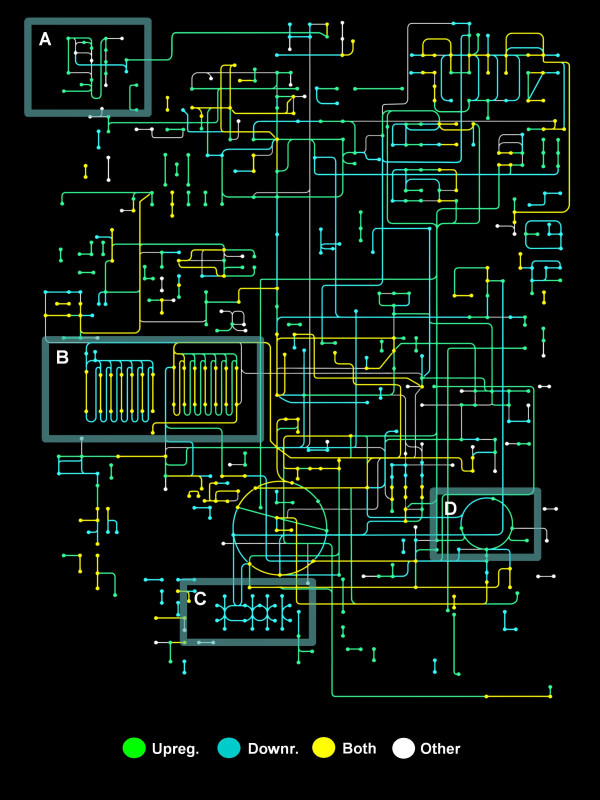
**Metabolic Atlas of Physarum polycephalum**. All *P. polycephalum *cDNAs (Refs [5,6] and our results) were sent for KEGG Ortholog [19] (KO) prediction using the KAAS server [20]. The output list of orthologs was used to plot this atlas with the KEGG mapping tool [21]. Nodes represent metabolites and edges (lines) correspond to enzymatic reactions. Colors are assigned to either down- (*light blue*) or upregulated (*green*) transcripts. Transcripts with equivalent relative frequencies in both novel cDNA libraries (relL/relD = 1) are also depicted (*yellow*); white represent those cDNAs with no expression data. After photoinduction, most enzymes from the N-glycan biosynthesis (A) and the urea cycle (D) pathways are upregulated. In contrast, cDNAs mapped to the oxidative phosphorylation (C) had higher relative frequencies in competent plasmodia, whereas a change from fatty acid synthesis to degradation is seen after photoinduction (B).

Functional annotation can also be classified using the pathway-based definition of ortholog genes from the Kyoto Encyclopedia of Genes and Genomes (KEGG) database [19]. In order to categorize the transcripts in KEGG pathways, we employed the KAAS server [20], a tool that uses similarity information to assign a sequence to a KEGG ortholog (KO) identifier, with default parameters for ESTs. We mapped 2,716 (16%) transcripts to 114 reference metabolic pathways, 1,904 including hit counts data, from which 770 correspond to cDNAs with higher relative frequencies in the library prepared from competent plasmodia, and 743 cDNAs in the library prepared from light-induced plasmodia respectively. In addition, 496 sequences in total were assigned to the KEGG BRITE hierarchies. Transcripts associated to the nucleotide metabolism (n = 110) and citrate cycle (n = 40) had the highest representation for the reference metabolic pathways, and the Wnt, TGF-beta and Jak- STAT signaling pathways were also depicted for the whole dataset (*n *= 49, 42 and 32 matches respectively). In the whole dataset we identified 420 cDNAs with potential roles in cell differentiation, with molecular entities associated to kinases (n = 140) and GTP binding (n = 110) having the highest representation in the BRITE hierarchies. In addition, we mapped 1,159 total enzyme commission (EC) numbers (418 unique) with 380 unique enzyme names in 851 transcripts, using the EC-module of BLAST2GO [16]. Later, in order to assess the global metabolic changes that occur after light induction, transcripts with KO identifiers were mapped using the KEGG Atlas tool [21]. For transcripts with higher relative frequencies in the competent plasmodia library, we mapped enzymes for the lipid biosynthesis (map00061) and oxidative phosphorylation (map00190) pathways. Conversely, we identified enzymes for the N-glycan biosynthesis (map00510), urea cycle (map00220) and fatty acid metabolism (map00071) pathways in transcripts with higher relative frequencies in the light-induced plasmodial library (Figure 2). In the end, we obtained 2,567 contigs annotated for GO terms, KEGG orthologs, and InterPro hits together [Additional File 7]. A summary of sequencing annotations and statistics is listed on the Table 1.

**Table 1:** Summary of the transcriptome sequencing and annotation.

Sequencing	
Total 454 reads	405,363

Total sequencing output (Mb)	61.9

Reads from the competent plasmodia library	125,456

Reads from the light-induced plasmodia library	99,632

**contigs**	

Total contigs	16,669

Contigs with hit counts	13,169

Contigs with at least 5 hits in both libraries	2,103

More abundant in the library from competent plasmodia	3,947^a^

More abundant in the library from light-induced plasmodia	4,972^b^

Downregulated, significant differential expression	1,149

Upregulated, significant differential expression	1,623

**similarity search**	

Total contigs with blastx results (e-value < 1E-3)	7,778

Contigs with blastx results and hit counts	5,544

Contigs with blastx results and significant differential expression	1,287

**annotations**	

Total contigs with GO annotations	3,304

Total contigs with KEGG orthologs	2,716

Total contigs with InterPro results	6,813

Contigs with GO annotations and hit counts	2,459

Contigs with KEGG orthologs and hit counts	1,904

Contigs with InterPro results and hit counts	5,180

^a ^Contigs with relative frequencies higher in the competent plasmodial library (*relD*/*relL *> 1), are classified as downregulated. Relative frequencies correspond to the number of hits per transcript divided by the total number of hits for each condition, competent (*relD*) and light-induced (*relL*) plasmodial libraries. ^b ^Upregulated transcripts: Those with relative frequencies higher in the light-induced plasmodial library (*relL*/*relD *> 1)

### Inference of Interaction Networks

**Figure 3:**
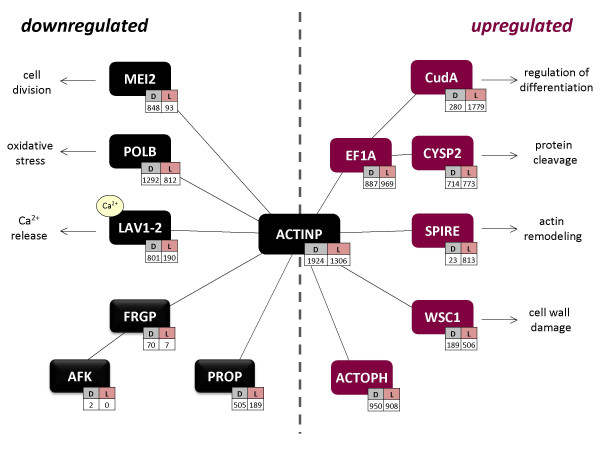
**Interactions with the Actin Cytoskeleton of Transcripts with Higher Relative Frequencies**. The network was hypothesized from interaction data reported in the literature, using transcripts previously clustered according to their relative frequencies (Figure 1 and Table S1). The transcripts shown are a subset of those from Figure 1, except for certain gene products (FRGP, AFK, and PROP) which were also included as their interactions have been previously observed in *Physarum*. cDNAs are displayed in colors corresponding to their expression status: down- (black) or up-regulated (red) upon photoinduction, as separated by the dotted vertical gray reference line. Each contig is shown with its hit number counts in both libraries (D: competent plasmodia, L: light-induced plasmodia).

In order to identify the functional relationships between the annotated cDNAs, we searched for known interactions in the literature. First, we used the cDNAs that were previously clustered according to their relative frequencies (Figure 1; [Additional File 5: Table S1]), and included additional proteins whose interactions have been observed in the literature for *Physarum*. Using the "guilt by association" heuristic to link coexpressed transcripts into functional groups [22,23], we inferred an interaction network between those transcripts. This network is primarily based on actin-binding activities (Figure 3).

Later, to identify genes with similar regulation, we listed those transcripts with highest rates of relative frequencies, counted in both cDNA libraries ([Additional File 8: Table S3]; [Additional File 9: Table S4]). As most of these highly differentially regulated transcripts did not show any sequence similarity to previously annotated genes, we clustered the subset of cDNAs with similarity to annotated genes according to two parameters: (*i*) their rate of relative frequencies; and (*ii*) their statistical significance of differential expression [9]. In this way we listed those 20 transcripts with annotations that were most up- or most downregulated in light-induced plasmodia, based on the statistical significance of their differential expression (P < 0.05; Tables 2 and 3). Despite the apparent diversity in biochemical functions, we searched for known interactions between these two groups of transcripts.

**Table 2:** Top 20 Annotated Transcripts Downregulated in Light-induced Plasmodia

Contig ID	SwissProt	Annotation	hits(D)	hits(L)	relD/relL	P-value
contig10338_1	P36618	Cell division control protein 16, CDC16	40	1	31.7301	7.48E-10

contig10470_1	P20072	Annexin A7	68	2	26.9706	1.54E-15

contig00397_1	Q5BMR2	Phospholipase D (PLD1)	62	2	24.5908	4.31E-14

contig00525_1	Q7EYV7	Poly ADP-ribose polymerase 1 (PARP-1)	244	10	19.3554	5.19E-49

contig11321_1	P38750	Uncharacterized transporter YHL008C	24	1	19.0381	5.43E-06

contig00901_1	P16064	Subtilisin inhibitor 1, ASI-I	21	1	16.6583	2.79E-05

PpolyN1a03a12	Q07346	Glutamate decarboxylase, GAD	20	1	15.8651	4.80E-05

PpolyN1a02c07	P34121	Coactosin, coaA	56	3	14.8074	1.07E-11

contig11574_1	P39749	Flap endonuclease 1, FEN-1	18	1	14.2786	0.000141

contig10414_1	Q5UNX2	Putative ankyrin repeat protein, YL715	90	5	14.2786	8.82E-18

contig03548_1	O49286	F-box/LRR-repeat protein 5, FBL5	17	1	13.4853	0.000242

contig00369_1	Q80U58	Pumilio homolog 2	17	1	13.4853	0.000242

contig10457_1	Q8WN03	Kv channel-interacting protein 2, Kcnip2	16	1	12.6921	0.000412

contig00264_1	P13466	Actin-binding protein 120, ABP-120	32	2	12.6921	5.26E-07

contig02333_1	Q8RWN7	POLTERGEIST Protein phosphatase 2C 32, PP2C	15	1	11.8988	0.000701

contig01650_1	O24496	Glyoxalase II, Glx II	15	1	11.8988	0.000701

contig01322_1	Q94B74	NADH pyrophosphatase	15	1	11.8988	0.000701

contig08310_1	Q10MW3	Pyruvate decarboxylase isozyme 2, PDC2	73	5	11.5815	6.84E-14

contig00558_1	P18281	Actobindin	29	2	11.5022	2.55E-06

contig11873_1	O10296	Apoptosis inhibitor 1, IAP-1	28	2	11.1055	4.30E-06

Transcripts with unambiguous annotations, significant differential expression (P < 0.05), and that possess the highest levels of downregulation (relD/relL > 1.0), are listed. BLAST2GO [15] automatic annotations were used, and manual corrections of annotations were included in some cases.

**Table 3:** Top 20 Annotated Transcripts Upregulated in Light-induced Plasmodia

Contig ID	SwissProt	Annotation	hits(D)	hits(L)	relL/relD	P-value
PpolyN1d39e07	O08623	Sequestosome-1, SQSTM1	3	171	71.6971	2.03E-56

contig02685_1	Q54IV3	ATP-dependent RNA helicase, DDX42	1	37	46.8103	7.87E-13

PpolyN1d106 h10	Q9U1K1	Spire	23	813	44.5504	5.40E-250

PpolyN1a08 g07	O08849	Regulator of G-protein signaling 2, RGS2	1	22	27.8276	9.99E-08

contig05590_1	Q8H100	ADP-ribosylation factor GTPase-activating, AGD8	1	21	26.5690	2.17E-07

PpolyN1a14d12	Q07283	Trichohyalin, TRHY	1	20	25.2931	4.69E-07

contig11781_1	Q55D99	Serine/threonine-protein kinase, pakA	2	34	21.5086	8.84E-11

contig06420_1	Q9UUG5	Myosin regulatory light chain 1, MLR1	1	17	21.5000	4.69E-06

contig08470_1	Q54MI7	Uncharacterized protein DDB_G0285917	1	16	20.2414	1.01E-05

contig12553_1	Q5R826	Transmembrane protein 63A, TM63A	20	308	19.4149	1.89E-83

PpolyN1d18d06	Q05924	Dosage-dependent cell cycle regulator 2, DCR2	1	15	18.9828	2.15E-05

contig08799_1	Q43207	Rotamase, FKBP70	1	14	17.7069	4.59E-05

contig12445_1	Q7S045	Non-histone chromosomal protein 6, NHP6	1	13	16.4483	9.76E-05

contig11110_1	P54678	Calcium-transporting ATPase, PAT1	1	13	16.4483	9.76E-05

contig08929_1	Q39572	Ras-related YPTC6	1	13	16.4483	9.76E-05

contig08360_1	Q6TQE1	Zinc finger- containing protein 18, NHN1	1	12	15.1724	2.06E-04

contig04102_1	Q9D0C1	Rab RING finger 7, RR7	4	47	14.8026	2.86E-13

contig03233_1	P06704	Cell division control protein 31, CDC31	2	23	14.5517	3.44E-07

contig02500_1	Q5UPW6	Putative FNIP repeat-containing protein, L281	2	23	14.5517	3.44E-07

contig08917_1	Q9PTW9	Proteasome subunit alpha type-7, PSMA7	1	11	13.9138	4.35E-04

A list of transcripts with unambiguous annotations, significant differential expression (P < 0.05) with the highest levels of upregulation (relL/relD > 1.0), is shown. Annotations, SwissProt accessions, hit counts, and probability values follow the same convention as in Table 2.

**Figure 4:**
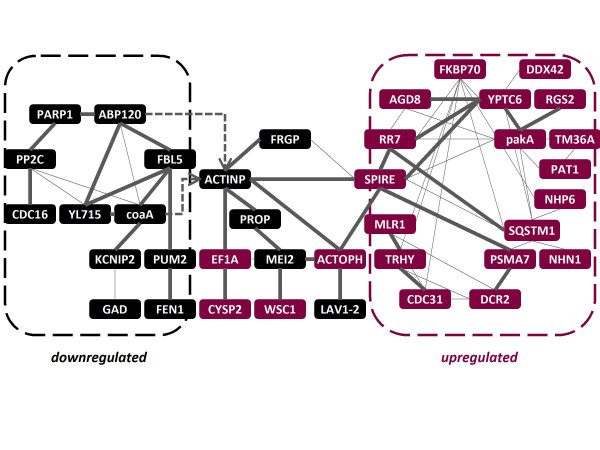
**Interaction of the Most Upregulated and Downregulated Transcripts with the Actin Cytoskeleton**. The conceptual network was predicted using the Cytoprophet module of Cytoscape [24-26], and therefore is solely based on information included on specialized interaction databases. Input transcripts included those from the top up- and down- regulated transcripts (Tables 2 and 3), and cDNAs taken from the previous interaction network (Figure 3). A significant probability of interaction (P-value > 0.9) is indicated as a thick edge. Node colors follow the same convention as in Figure 3, and hit count data can be found in Tables 2 and 3. This network includes 64 interactions (33 with P > 0.9) between 38 gene products. Genes without Cytoprophet-predicted interactions are not included, except for two interactions with Actin-P that were not predicted by Cytoprophet but that can be found in the literature (indicated with arrows). Interestingly, the previously featured network (Figure 3) connects the two groups of up- and downregulated transcripts in this figure. However, as Cytoprophet gathers experimental interaction data from specialized databases, some interactions depicted in Figure 3 are not shown (for example between POLB and ACTINP), because this data is not present on those source databases used by Cytoprophet for prediction.

From annotations of the top up- and down-regulated transcripts (Tables 2 and 3), and including the transcripts from the above mentioned analysis (Figure 3), we extended the initial putative network using Cytoprophet [24]. This Cytoscape [25] plugin predicts networks based on information from interaction databases, associated to SwissProt matches of newly annotated genes [26]. Accordingly, we found that most of these genes encoded proteins predicted to interact in a network of actin-binding proteins (coaA, ABP120, actobindin, FRGP, AFK, PROP; Figure 4). These genes encoding proteins orthologs of which are associated to cell division (MEI2, PUM2, CDC16), DNA repair (POLB, FEN1), signal transduction (PP2C, CDC16), and calcium-binding (LAV1-2, KCNIP2, GAD) are downregulated in light-induced plasmodia (Table 2; [Additional File 5: Table S1]). In turn, a different group of developmentally regulated genes is preferentially expressed after photoinduction, including genes the products of which are involved in signaling (DCR2, RGS2, YPTC6, pakA), protein processing (FKBP70, sequestosome-1, PSMA7, RR7), cell integrity (WSC1, CDC31), calcium-binding (MLR1, TRHY, PAT1), and developmentally regulated actin-binding, such as the elongation factor 1α (EF1A), spire, and actophorin (Table 3; [Additional File 5: Table S1]; Figures 3 and 4).

## Discussion

The development of plasmodia competent for sporulation includes growth arrest, condensation of cellular constituents, and mitosis [27]. Sporulation of competent plasmodia can be triggered by a light pulse. Some proteins associated with the light-induced pathways that lead to sporulation have been described [2-4], suggesting that several signaling mechanisms are involved, but there are no studies that describe changes at the level of the whole transcriptome. In the present study we identified the most up- and downregulated transcripts, which are associated to a network of putative interactions (Figure 4). The network is hypothetical as interactions used for inference are based on data obtained from different organisms. For the sake of simplicity, the discussion will be focused on genes with predicted significant interactions (P > 0.9).

### A network of actin-binding proteins is associated to changes during light-induced sporulation in Physarum

The actin cytoskeleton of *Physarum *is essential for locomotion, division, and other biological processes [27]. Assembly and disassembly of actin filaments is controlled by a group of actin-binding proteins, whose activities in turn are regulated by specific signaling pathways. *Physarum *cell types differ in actin organization but express the same actin genes, suggesting that changes in actin-binding proteins are responsible for the differences in actin organization [28]. *Physarum *possesses several classes of actin- binding proteins, and most of these proteins display cell type-specific patterns of expression, but their precise roles are not known [29,30]. Nevertheless, expression changes in genes coding for actin-binding proteins correlate with modifications in cell organization and behavior [27]. In the present study, we found that some actin-binding genes were linked specifically to stages before and after photoinduction in the starved *Physarum *plasmodium.

Specifically, we identified protist orthologs for actin-binding proteins, including *Dictyostelium *coaA (Coactosin A [31]) and ABP-120 (actin-binding protein 120), and actobindin from *Acanthamoeba*, which binds actin monomers (Table 2) [32]. Coactosin A interferes with the capping of F-actin filaments [33], and is differentially expressed after metal exposure in worms [34]. ABP-120 organizes filamentous actin into networks of fibers, and *Dictyostelium *cells lacking ABP-120 have a severe phototaxis defect at the multicellular slug stage [35]. In addition, we noticed that transcripts coding for *Physarum *plasmodia-specific actin-binding proteins, such as profilin P (PROP) [30] and fragmin P (FRGP) [36], are downregulated after photoinduction (Figure 3). FRGP enables actin phosphorylation by the actin-fragmin kinase (AFK), and binds phosphorylated actin [29,36]. Therefore it is possible that during sporulation these proteins are involved in the reorganization of the subcellular compartments via interactions with the actin cytoskeleton.

### Transcripts linked to cell division and DNA repair are downregulated in the light-induced plasmodium

After several days of starvation, cell processes must be limited in order to save energy. Coordination of several biological processes is then required, and thus regulation of these phenomena needs a pleiotropic transducer like the cAMP, which targets several signaling pathways, including those that limit cell proliferation [37]. Cell differentiation pathways regulated by cAMP levels have been described in *Dictyostelium*, a closely related protist [38]. For *Physarum*, we found that MEI2, a transcript controlled via cAMP levels, is downregulated in the light-induced plasmodium (Figure 3; [Additional File 5: Table S1]). MEI2 is an RNA-binding protein that encodes a cAMP-regulated positive regulator of meiosis in the yeast *S.pombe *[39,40]. This gene product is functionally related to the actin cytoskeleton via the cAMP-dependent protein kinase A (PKA) [37,38]. Other transcripts downregulated in light-induced plasmodia associated to cell division and DNA repair comprised FEN1, CDC16 and PUM2. First, the Flap endonuclease 1 (FEN1) appears in several processes linked to the maintenance of the genome integrity, such as the UV-induced DNA repair [41], as well as in DNA replication and DNA recombination [42]. Second, the yeast cell division control protein 16 (CDC16), constitutes the catalytic subunit of the spg1p GTPase-activating protein, that is involved in the signal transduction controlling septum formation. CDC16 is involved in cytokinesis [43] and is essential for proliferation, as spores lacking a functional CDC16 gene complete mitosis without undergoing cell cleavage [44]. Finally, PUM2 (Pumilio 2) encodes a RNA-binding protein associated to the control of meiosis during development [45]. Consequently, starvation seems to be the signal that regulates cell division while protecting the cells from oxidative stress, through cAMP-regulated pathways (Figure 3).

Other downregulated transcripts in the light-induced plasmodium comprised orthologs of transducers, such as FBL5, a leucine-repeat protein linked to phosphorylation-dependent ubiquitination [46], PARP1, an *Oryza *poly ADP-ribose polymerase, a phospholipase D from *Phytophtora *(PLD1), and the *Arabidopsis *phosphatase 2C (PP2C, also known as Poltergeist). In plants, G-proteins are involved in phospholipase D activation, and this also seems to be the case for *Phytophtora *[47]; on the other hand, PP2C operates in several signaling pathways that regulate stem cell differentiation [48]. It is then reasonable to consider that the differential expression of these transducers is also associated with the control of signaling mechanisms for differentiation, but more profound studies are needed to establish precise causal relationships.

### Calcium- binding proteins exhibit diverse regulation patterns in the light-induced plasmodium

Transcripts identified as calcium-binding proteins displayed different patterns of expression regulation. These were either down- (LAV1-2, KCNIP2 and GAD) or upregulated (MLR1, TRHY, and PAT1) after light induction. LAV1-2 is a plasmodium-specific RNA of unknown function that encodes a protein containing an EF-hand type domain whose calcium-binding activity has been observed *in vitro *in *Physarum *[49]. LAV1-2 seems to act as a sensor of cell damage, releasing Ca^2+ ^that leads to the activation of a plasmodium-specific transglutaminase, which separates damaged areas of a plasmodium [50]. Other transcripts encoding orthologs of calcium-binding proteins, such as KCNIP2 and GAD, were also downregulated in the photoinduced plasmodium and have not been previously described for in *Physarum*. KCNIP2 encodes a potassium channel-interacting protein that probably modulates channels density in a Ca^2+^- dependent manner. In turn, the activation of glutamate decarboxylase (GAD) by calcium-bound calmodulin (CaM) is required for normal growth in plants [51]. Previous studies have shown that the intracellular increase of calcium levels is correlated with increased concentrations of cAMP and with sporulation and differentiation in both *Physarum *and *Dictyostelium *[52,53]. Moreover, actin filament crosslinking is affected by changes in intracellular calcium levels, which ultimately influences the cell contractility [54]. Therefore it seems possible that these calcium-binding proteins coordinate the Ca^2+ ^release as a means to influence the cell contractility through the interaction with the actin cytoskeleton (Figure 4).

Furthermore, the upregulated subset of calcium-binding proteins included MLR1, which inhibits cytokinesis in yeasts; trychohyalin (TRHY), which is involved in its own calcium-dependent processing during differentiation; and the *Dictyostelium *PAT1 ATPase. PAT1 is localized in the membrane of contractile vacuoles, and is a component of a calcium sequestration and excretion pathway, which functions to help maintain homeostasis, especially under conditions of Ca^2+ ^stress [55]. Thus these are candidates to control the intracellular calcium levels after light induction of starved plasmodia.

### Actin-binding proteins associated to development are overexpressed in the light-induced plasmodium

After photoinduction, a group of actin-binding proteins is upregulated including the elongation factor 1α (EF1A), Spire, and actophorin (Figures 3 and 4; Table 3; [Additional File 5: Table S1]). Spire is a *Drosophila *gene involved in development through actin assembly. This gene is also widely distributed across the metazoan genomes. Spire mammalian isoforms are MAP kinase substrates, and data suggest that Spire evolved as an alternative independent mechanism of actin polymerization, necessary for cell polarization in multicellular organisms [56]. Actophorin, in turn, binds actin monomers and separates actin filaments in a dose-dependent manner. Phosphorylation of actophorin blocks actin binding [57]. In turn, EF1A, aside from its role in the protein synthesis, has a separate conserved actin-binding activity in eukaryota, initially observed in *Dictyostelium *[58], where it is predominantly found in actin-bound form [59]. EF1A regulates the stoichiometry of cytoskeletal components, and the conservation of the EF1A-actin interaction across eukaryotes suggests its importance for cytoskeletal maintenance [60]. Overexpression of EF1A in yeast results in effects on cell growth, and influences the actin distribution, morphology and budding in a dosage-dependent manner, although this increase of EF1A has no effect over the protein synthesis [61]. In addition, changes in cytoskeletal redistribution of EF1A seem to be linked to the differentiation status, where the association between EF1A and microtubules gradually increases in differentiating cultures [62]. Furthermore, EF1A stimulates actin remodeling and induces the formation of filopodia [63], and possibly connects these processes with signaling pathways [64].

We noticed that two coexpressed transcripts (the cysteine proteinase CYSP2 and the developmentally regulated gene CudA) are related to EF1A. First, cysteine proteinases are believed to participate in protein cleavage during the differentiation of *Dictyostelium *as a response to starvation [65], and these peptidases were copurified with EF1A in yeasts [66]. CudA, on the other hand, is associated to the transition from slug migration to culmination in *Dictyostelium*. CudA expression levels depend on local cAMP concentration [67]. Recent evidences show that CudA contains a novel DNA-binding site that is distantly related to the metazoan STAT domains, which participate in the regulation of developmentally controlled genes [68], and whose orthologs coexpress with EF1A [69]. Yamada et al. [68] also proved a relationship between *Dictyostelium *CudA and a cDNA from *Physarum*, which corresponds to the contig reported here as a CudA ortholog. For these reasons, EF1A could work as a link between regulation of the protein synthesis, cytoskeletal maintenance, and signal transduction in slime molds (Figure 3).

Other developmentally regulated transcripts associated to the actin cytoskeleton included the cell wall integrity and stress response component (WSC1), which is a yeast membrane protein that acts as a sensor of cell wall damage [70], and CDC31, a constituent of the nuclear pore complex that is also involved in the maintenance of cell morphology (Table 3 and Figure 4). WSC1 is essential to keep the cell integrity, behaving like a stress-specific signal transducer that is involved in the reorganization of the actin cytoskeleton in response to osmotic shock [71,72]. WSC1 is involved in the depolarization of the actin cytoskeleton [72], and, like CDC16 (downregulated in light-induced plasmodia), is entailed in cytokinesis [73].

### GTP signaling genes involved in different processes are upregulated in the light-induced plasmodium

Orthologs of certain genes highly upregulated in light-induced plasmodia are involved in signal transduction. These include transcripts linked to the GTP signaling (AGD8, YPTC6, RGS2), kinases (pakA) and phosphatases (DCR2). The serine/threonine-kinase pakA is a regulator of the myosin component of the cytoskeleton, required for cytokinesis and the regulation of the cytoskeleton during chemotaxis in *Dictyostelium *[74]. In turn, the yeast dosage-dependent cell cycle regulator 2 (DCR2), is a phosphatase whose increased dosage alters cell cycle progression, while its loss delays the progression in the G1 phase [75]. In addition, upregulated GTP signaling transducers included a putative GTPase- activating protein from Arabidopsis (AGD8); a *Chlamydomonas *GTP-binding protein (YPTC6); and RGS2, which acts as a negative regulator of G-protein signaling, a function that is evolutionarily conserved in yeast, *C. elegans *and mammals. Increased RGS2 expression is primarily mediated by the cAMP/PKA pathway [76], therefore it is possible that RGS2 is carrying out similar tasks in slime molds, where it could work in coordination with the other transducers, as hypothesized in Figure 4.

### Transcripts annotated for cell death are overrepresented in the light-induced plasmodium

Comparison of GO terms between up- and downregulated groups showed that transcripts annotated for 'cell development' (GO:0048468), 'cell death' (GO:0008219) and 'death' (GO:0016265) were overrepresented in the upregulated group [Additional File 6: Table S2]. However, all these ontologies belong to the same hierarchy, meaning that 'cell death' can be the product of either development or organismal death, and hence 'cell death' is the only difference between both expression groups. One of these cDNAs annotated for 'cell death' is Sequestosome 1, which is also included on the list of upregulated transcripts (Table 3). Sequestosome 1, also known as p62, is a multifunctional protein that targets polyubiquitinated proteins to degradation by proteasomes and autophagy [77]. p62 knockouts significantly increased cell death [78], and this is probably linked to the interaction with atypical protein kinase C isoforms that are involved in pathways that control differentiation and apoptosis [79]. Therefore it is likely that this gene product regulates cell death pathways linked to the commitment for sporulation.

Furthermore, other highly upregulated genes are also functionally linked to the protein turnover. These include the FKBP70 rotamase, which accelerates the folding of proteins during synthesis; the PSMA7 proteasome subunit, which together with the other subunits, suffer changes during the meiotic cell cycle [80]; and the endosome-lysosome vesicle traffic-related RR7 [81]. It is likely then that these gene products, together with Sequestosome 1, are linked to the control of differentiation through post-transcriptional regulation.

## Conclusions

The gain of sporulation-competence of *Physarum *plasmodia involves growth arrest, condensation of constituents, and mitosis and is a prerequisite before sporulation can be induced by light [27]. *Physarum *gene expression has been shown to be cell type-specific, but existing studies have been focused only on individual genes [2-4]. Previously, we reported a library of 5,856 sequences obtained from plasmodia competent for the induction of sporulation [6]. In the present study we used the massive parallel sequencing technology at the level of the whole transcriptome [7,8] in order to identify global changes in expression that occur during light-induced sporulation of *Physarum*. We integrated the differentially expressed cDNAs into networks using interaction information from orthologs and the literature. We found that after light induction of a plasmodium the expression of transcripts linked to cell division and DNA repair is downregulated. In contrast, light-induction stimulated the expression of genes associated with protein turnover (proteases and proteasome transcripts), genes related to cell cycle progression, and genes involved in the maintenance of cell integrity and cytokinesis. These latter gene products might protect the cell against osmotic shock. Additionally, different groups of calcium-binding proteins are either down- or upregulated after light exposure. These gene products are candidates to control the intracellular calcium levels during sporulation. We postulate that these changes are associated with a network of actin-binding proteins (Figures 3 and 4), the components of which are differentially regulated upon plasmodial photoinduction. It seems that these gene products accomplish different tasks in each stage: the reorganization of the subcellular compartments in order to inhibit migration during starvation on one hand, and cell polarization and cytoskeletal redistribution after photoinduction mediated by a group of actin-binding proteins on the other. We expect that the precise representation of the proposed interaction networks may become available as gene knockout experiments, proteomic data, and comparative interactomics are integrated in future studies of this organism.

## Methods

### Culture and light-induction of plasmodial cells

*Physarum *plasmodia of the white strain (LU897 × LU898) were hatched from spherules, and grown as microplasmodial suspensions for four days. The plasmodial mass was then applied to starvation agar plates. Microplasmodia spontaneously fused to give a single plasmodium on each plate. Plasmodia were then starved for six days in the dark at 22°C to obtain maximal competence for sporulation. To verify the sporulation-competent state, plasmodia were cut into two halves. One half was immediately frozen in liquid nitrogen for RNA extraction, and the other half was returned to the dark and incubated until the next day to verify that the plasmodium had not been induced to sporulation. To obtain light-induced plasmodia, competent plasmodia were irradiated for 30 min with far red light and then returned to the dark. Six hours after the start of irradiation, plasmodia were cut into two halves. One half was frozen in liquid nitrogen for RNA extraction. The other half was returned to the dark and incubated until the next day to verify the sporulation status [3,4].

### cDNA Library Construction and Sequencing

Transcript poly(A)^+ ^RNAs were isolated by oligo-dT chromatography. cDNAs were prepared from these RNAs by the full-length enriched synthesis method (vertis Biotechnologie, Freising- Weihenstephan, Germany). First strand cDNA was synthesized using oligo(dT) adapter primers and MMLV H-reverse transcriptase. Following RNA hydrolysis, an adapter primer was annealed to the 3' end, and the produced fragments were PCR-amplified for 22 cycles with a proofreading enzyme. The cDNA libraries were then directly sequenced using the 454 GS FLX system (Roche Diagnostics, Mannheim, Germany) [7]. Chromatograms were scored for quality, and the produced sequences were trimmed of adapter sequences, and coassembled into contigs using previously available transcriptomic data [6]. For expression comparisons we obtained for each contig: (*i*) the number of reads (which we define as "hit counts") in both libraries; (*ii*), their relative frequencies (reads of a given contig divided by the total number of reads); and (*iii*) their relative frequencies. Statistical significance between the two hit counts for each contig species was then assessed [9].

### Sequence Annotation and Network Inference

Similarity searches against protein databases were performed using BLASTX implemented in the MIGenAS tool [82] (e-value 1 × 10-3). We utilized nine protein databases in this comparison: Swiss-Prot and TrEMBL (versions 56.3 and 39.3) [12], dictyBase [14] and RefSeq database subsets: mammalian, other vertebrates, invertebrate, protozoa, plant and microbial (release 31) [13]. Functional annotation was carried out using BLAST2GO (version 2.2.3) [16]. This procedure consisted of a similarity search against the non-redundant GenBank database [10], using BLASTX (e-value 1 × 10-3), followed by Gene Ontology (GO) [15] mappings extracted from similarity results and InterPro domain matches (InterPro release 18.0) [17]. Annotation of sequences (cutoff value 1 × 10-6) was followed by their validation, and these annotations were extended using ANNEX [83]. Statistical analysis of GO annotations between differentially expressed cDNAs was carried out using the Fisher exact test, as implemented in the GOSSIP module [18] of BLAST2GO. Sequences were also categorized in metabolic and signaling pathways, via similarity search against orthologs present in the KEGG database [19] using the KAAS server [20]. In this case, we employed default parameters for ESTs. KEGG orthologs (KOs) were then plotted into the whole metabolic atlas, utilizing the KEGG mapping tool [21]. Putative networks of correlated genetic interactions were generated from annotation information, using the MLE algorithm [26], as implemented in the Cytoprophet plugin [24] of Cytoscape [25].

## Abbreviations

EF1A: elongation factor 1- alpha; WSC1: cell wall integrity and stress response component; mei2: yeast meiosis- related protein; PKA: cAMP- dependent protein kinase A; STAT: Signal transducer and activator of transcription; GO: Gene Ontology; FDR: false discovery rate; KEGG: Kyoto Encyclopedia of Genes and Genomes; KAAS: KEGG Automatic Annotation Server; KO: KEGG ortholog.

## Authors' contributions

WM conceived of the research. GG and WM designed the study, and IB contributed to the experimental design. WM cultured the cells, isolated RNA, and coordinated the project. GG performed the sequencings, contig assemblies and quantitations. SM and IB carried out statistical analyses and performed similarity searches. IB annotated the transcriptome, as well as modeled interaction networks. IB wrote the paper, and WM and GG helped with the manuscript preparation. All authors read and approved the final manuscript.

## Authors' Information

Website for Affiliation 5: http://www.ovgu.de/ag-marwan/

## Supplementary Material

Additional file 1 **Figure S1**. Overview of the Experimental Design. A summary of experiments and computational analyses is depicted. RNA samples were taken from competent plasmodia after six days of starvation in the dark, and from competent plasmodia at six hours after exposition to a 30 minutes pulse of red light (≥ 700 nm) (*1*) [3]. cDNAs were synthesized from extracted RNAs (*2*), and sequenced and quantitated using the 454 Life Sciences platform (*3*) [7,8]. Contigs generated were then annotated at every bioinformatic level (*4*), and network interactions (*5*) were obtained both by a combination of manual curation of literature, expression data, and predictions from annotations (Powerpoint file). Click here for file

Additional file 2 **Transcript Abundance Data**. Hit counts obtained from pyrosequencing, including statistical analyses (Excel spreadsheet). Click here for file

Additional file 3 **Figure S2. Hits Distribution of Transcript Species**. The distribution of pyrosequencing hit counts respect to the number of transcript species on each library (starvation and light-induced) is depicted on a semi-logarithmic scale. Hit counts are included in the adjacent upper ranges to the right; for example, transcripts with 2 hits are present in the 2-5 range. Similar distributions of contig species were found on both libraries, and most transcripts were represented by 1 to 5 hits only (Powerpoint file). Click here for file

Additional file 4 **Similarity Results**. Results of the similarity analyses against protein databases from sequences with hit counts data (Excel spreadsheet). Click here for file

Additional file 5 **Table S1. Annotated transcripts with relative frequencies higher than 0.005**. A list of transcripts obtained from the scatterplot of relative frequencies (Figure 1) is depicted. Annotations, hit counts, and probability values follow the same convention as in Table 2 (Word document). Click here for file

Additional file 6 **Table S2. Overrepresented Gene Ontology terms in Upregulated Transcripts**. Full lists of GO terms from up- and downregulated contigs were compared against each other using the Fisher's exact test from the GOSSIP program [18], as implemented in BLAST2GO [16]. A two-tailed test with the false discovery rate (*FDR*) filter was employed. The number of GO-annotated transcripts used for comparison between up- (*Test*) and downregulated (*Ref*) groups of cDNAs is shown. All overrepresented GO terms belong to the biological process (BP) category (Word document). Click here for file

Additional file 7 **Transcriptome Annotation**. Annotations of all sequences, including those with or without hit counts data (Excel spreadsheet). Click here for file

Additional file 8 **Table S3. Top 20 Transcripts Downregulated in Light-induced Plasmodia**. Transcripts with the highest rates of downregulation (relD/relL > 1.0), are listed. BLAST2GO [15] automatic annotations were used, and manual corrections were included in some cases. Transcripts with unknown orthologs are described with "---NA---." Annotations, SwissProt accessions, hit counts, and probability values follow the same convention as in Table 2 (Word document). Click here for file

Additional file 9 **Table S4. Top 20 Transcripts Upregulated in Light-induced Plasmodia**. Transcripts with the highest rates of upregulation (relL/relD > 1.0), are listed. BLAST2GO [15] automatic annotations were used, and manual corrections were included in some cases. Columns follow the same convention as in Table S3 (Word document). Click here for file
