# Supplementary figures and images for: Transcriptomic changes arising during light-induced sporulation in Physarum polycephalum
Source: BMC Genomics. 2010 Feb 17;11:115. doi: 10.1186/1471-2164-11-115 (PMC2837032; doi:10.1186/1471-2164-11-115)

## Slide 1
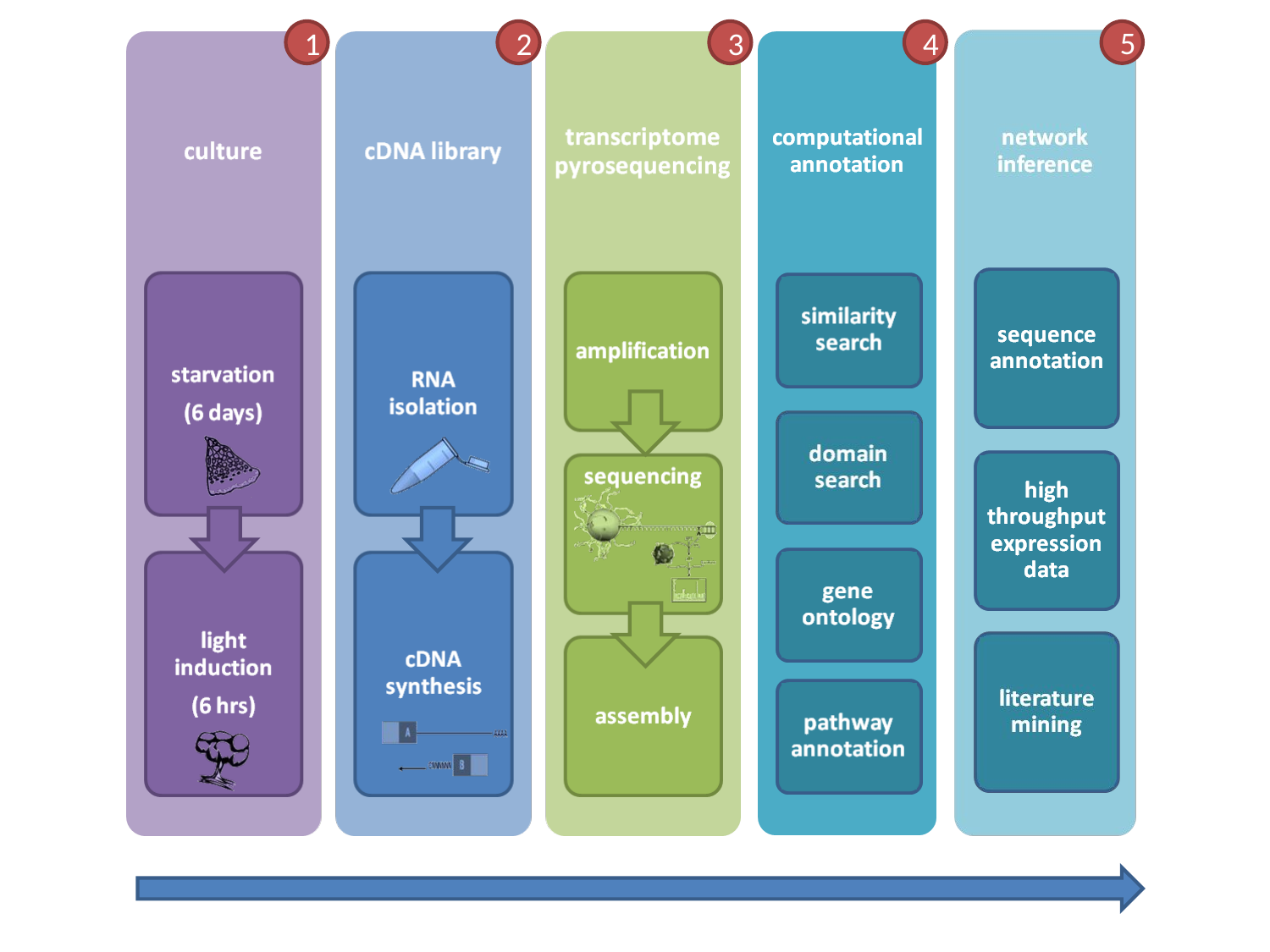

1
2
3
4
5

Supplement: Additional file 1 — Figure S1. Overview of the Experimental Design. A summary of experiments and computational analyses is depicted. RNA samples were taken from competent plasmodia after six days of starvation in the dark, and from competent plasmodia at six hours after exposition to a 30 minutes pulse of red light (≥ 700 nm) (1) [3]. cDNAs were synthesized from extracted RNAs (2), and sequenced and quantitated using the 454 Life Sciences platform (3) [7,8]. Contigs generated were then annotated at every bioinformatic level (4), and network interactions (5) were obtained both by a combination of manual curation of literature, expression data, and predictions from annotations (Powerpoint file). [file 1471-2164-11-115-S1.ppt]

## Slide 1
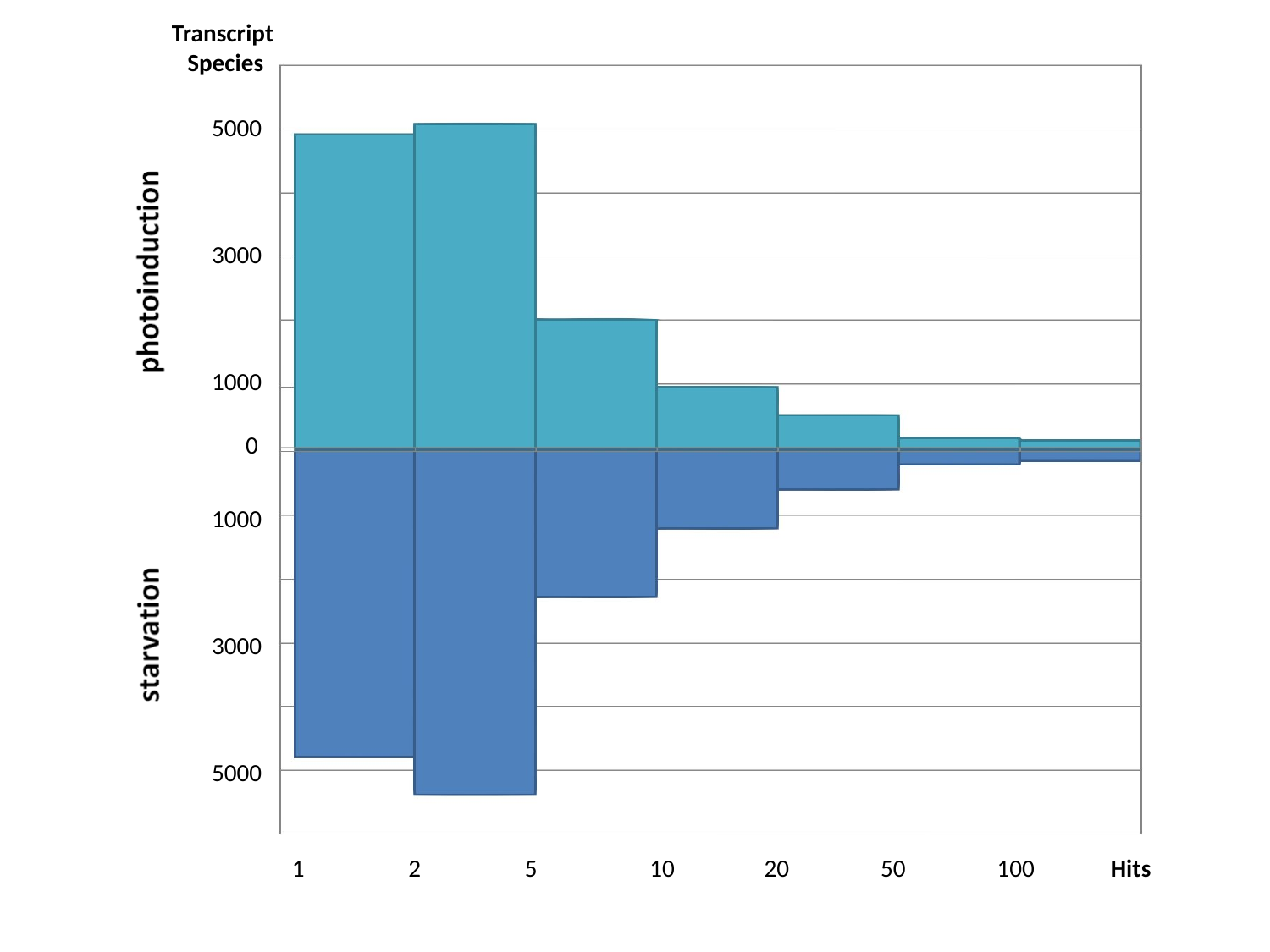

Transcript
Species
5000
3000
1000
0
1000
3000
5000
1
2
5
10
20
50
100
Hits

Supplement: Additional file 3 — Figure S2. Hits Distribution of Transcript Species. The distribution of pyrosequencing hit counts respect to the number of transcript species on each library (starvation and light-induced) is depicted on a semi-logarithmic scale. Hit counts are included in the adjacent upper ranges to the right; for example, transcripts with 2 hits are present in the 2-5 range. Similar distributions of contig species were found on both libraries, and most transcripts were represented by 1 to 5 hits only (Powerpoint file). [file 1471-2164-11-115-S3.ppt]
