# Supplementary material for: Transcriptomic changes arising during light-induced sporulation in Physarum polycephalum
Source: BMC Genomics. 2010 Feb 17;11:115. doi: 10.1186/1471-2164-11-115 (PMC2837032; doi:10.1186/1471-2164-11-115)
Supplement: Additional file 5 — Table S1. Annotated transcripts with relative frequencies higher than 0.005. A list of transcripts obtained from the scatterplot of relative frequencies (Figure 1) is depicted. Annotations, hit counts, and probability values follow the same convention as in Table 2 (Word document). [file 1471-2164-11-115-S5.doc]

| **Contig ID** | **Annotation** | **hits(D)** | **hits(L)** | **P-value** |
| --- | --- | --- | --- | --- |
| PpolyN1d50g09 | Transcriptional Regulator CudA | 280 | 1779 | 0.00 |
| PpolyN1d38e09 | Elongation Factor 1-alpha, EF1A | 887 | 969 | 3.31E-12 |
| contig04302_1 | Actophorin | 950 | 908 | 3.33E-05 |
| contig12806_1 | Cysteine Proteinase 2, CYSP2 | 714 | 773 | 1.17E-09 |
| contig04331_1 | Cell wall integrity and stress response component, WSC1 | 189 | 506 | 1.03E-52 |
| PpolyN1d106h10 | Spire | 23 | 813 | 5.38E-250 |
| contig12440_1 | DNA Polymerase beta, POLB | 1292 | 812 | 7.17E-08 |
| PpolyN0a10e04 | Plasmodial-specific protein LAV1-2 | 801 | 190 | 1.74E-62 |
| PpolyN1d32d11 | Meiosis protein MEI2 | 848 | 93 | 1.26E-118 |
| PpolyN0a11e12 | Actin P, plasmodial isoform | 1924 | 1306 | 5.56E-06 |
