# Supplementary material for: Transcriptomic changes arising during light-induced sporulation in Physarum polycephalum
Source: BMC Genomics. 2010 Feb 17;11:115. doi: 10.1186/1471-2164-11-115 (PMC2837032; doi:10.1186/1471-2164-11-115)
Supplement: Additional file 6 — Table S2. Overrepresented Gene Ontology terms in Upregulated Transcripts. Full lists of GO terms from up- and downregulated contigs were compared against each other using the Fisher's exact test from the GOSSIP program [18], as implemented in BLAST2GO [16]. A two-tailed test with the false discovery rate (FDR) filter was employed. The number of GO-annotated transcripts used for comparison between up- (Test) and downregulated (Ref) groups of cDNAs is shown. All overrepresented GO terms belong to the biological process (BP) category (Word document). [file 1471-2164-11-115-S6.doc]

| **GO term** | **GO description** | **FDR** | ***P*-value** | **Test** | **Ref** |
| --- | --- | --- | --- | --- | --- |
| GO:0048468 | cell development | 0.009272 | 0.000314 | 35 | 8 |
| GO:0008219 | cell death | 0.009272 | 0.000314 | 35 | 8 |
| GO:0016265 | death | 0.009272 | 0.000314 | 35 | 8 |
