# Supplementary material for: Transcriptomic changes arising during light-induced sporulation in Physarum polycephalum
Source: BMC Genomics. 2010 Feb 17;11:115. doi: 10.1186/1471-2164-11-115 (PMC2837032; doi:10.1186/1471-2164-11-115)
Supplement: Additional file 8 — Table S3. Top 20 Transcripts Downregulated in Light-induced Plasmodia. Transcripts with the highest rates of downregulation (relD/relL > 1.0), are listed. BLAST2GO [15] automatic annotations were used, and manual corrections were included in some cases. Transcripts with unknown orthologs are described with "---NA---." Annotations, SwissProt accessions, hit counts, and probability values follow the same convention as in Table 2 (Word document). [file 1471-2164-11-115-S8.doc]

| **Contig ID** | **SwissProt** | **Annotation** | **hits(D)** | **hits(L)** | **relD/relL** | **P-value** |
| --- | --- | --- | --- | --- | --- | --- |
| contig12399_1 | ---NA--- | ---NA--- | 368 | 3 | 97.30570 | 1.58E-88 |
| contig12495_1 | ---NA--- | ---NA--- | 141 | 2 | 55.92433 | 1.84E-33 |
| contig00052_1 | ---NA--- | ---NA--- | 45 | 1 | 35.69638 | 4.49E-11 |
| contig10338_1 | P36618 | Cell division control protein 16, CDC16 | 40 | 1 | 31.73012 | 7.48E-10 |
| contig10470_1 | P20072 | Annexin A7 | 68 | 2 | 26.97060 | 1.54E-15 |
| contig00397_1 | Q5BMR2 | Phospholipase D, PLD | 62 | 2 | 24.59084 | 4.31E-14 |
| contig01934_1 | ---NA--- | ---NA--- | 27 | 1 | 21.41783 | 1.04E-06 |
| PpolyN0a05b03 | ---NA--- | ---NA--- | 50 | 2 | 19.83132 | 3.20E-11 |
| contig00525_1 | Q7EYV7 | Poly [ADP-ribose] polymerase 1, PARP1 | 244 | 10 | 19.35537 | 5.19E-49 |
| contig11321_1 | P38750 | Uncharacterized transporter YHL008C | 24 | 1 | 19.03807 | 5.43E-06 |
| contig03338_1 | ---NA--- | ---NA--- | 23 | 1 | 18.24482 | 9.39E-06 |
| contig02945_1 | ---NA--- | ---NA--- | 22 | 1 | 17.45157 | 1.62E-05 |
| contig02169_1 | ---NA--- | ---NA--- | 22 | 1 | 17.45157 | 1.62E-05 |
| contig00994_1 | ---NA--- | ---NA--- | 22 | 1 | 17.45157 | 1.62E-05 |
| PpolyNOa14b03 | ---NA--- | ---NA--- | 151 | 7 | 17.11160 | 4.08E-30 |
| contig00901_1 | P16064 | Subtilisin inhibitor 1 | 21 | 1 | 16.65831 | 2.79E-05 |
| PpolyN1a03a12 | Q07346 | Glutamate decarboxylase, GAD | 20 | 1 | 15.86506 | 4.80E-05 |
| contig00391_1 | ---NA--- | ---NA--- | 20 | 1 | 15.86506 | 4.80E-05 |
| PpolyN1a02c07 | P34121 | Coactosin, coaA | 56 | 3 | 14.80739 | 1.07E-11 |
| contig00477_1 | ---NA--- | ---NA--- | 110 | 6 | 14.54297 | 1.67E-21 |
