# Supplementary material for: Transcriptomic changes arising during light-induced sporulation in Physarum polycephalum
Source: BMC Genomics. 2010 Feb 17;11:115. doi: 10.1186/1471-2164-11-115 (PMC2837032; doi:10.1186/1471-2164-11-115)
Supplement: Additional file 9 — Table S4. Top 20 Transcripts Upregulated in Light-induced Plasmodia. Transcripts with the highest rates of upregulation (relL/relD > 1.0), are listed. BLAST2GO [15] automatic annotations were used, and manual corrections were included in some cases. Columns follow the same convention as in Table S3 (Word document). [file 1471-2164-11-115-S9.doc]

| **Contig ID** | **SwissProt** | **Annotation** | **hits(D)** | **hits(L)** | **relD/relL** | **P-value** |
| --- | --- | --- | --- | --- | --- | --- |
| contig10367_1 | ---NA--- | ---NA--- | 1 | 79 | 99.94828 | 2.20E-27 |
| PpolyN0a10a04 | ---NA--- | ---NA--- | 9 | 565 | 79.11450 | 4.30E-184 |
| PpolyN1d39e07 | O08623 | Sequestosome 1 | 3 | 171 | 71.69714 | 2.03E-56 |
| contig00236_1 | ---NA--- | ---NA--- | 1 | 54 | 68.31034 | 1.08E-18 |
| contig12905_1 | ---NA--- | ---NA--- | 2 | 82 | 51.87069 | 4.73E-27 |
| contig01485_1 | ---NA--- | ---NA--- | 1 | 41 | 51.86207 | 3.32E-14 |
| contig12498_1 | ---NA--- | ---NA--- | 10 | 402 | 50.68041 | 2.39E-126 |
| contig02685_1 | Q54IV3 | ATP-dependent RNA helicase DDX42 | 1 | 37 | 46.81034 | 7.87E-13 |
| PpolyN1d106h10 | Q9U1K1 | Protein Spire | 23 | 813 | 44.55041 | 5.38E-250 |
| contig11969_1 | ---NA--- | ---NA--- | 1 | 30 | 37.94828 | 1.95E-10 |
| contig03550_1 | ---NA--- | ---NA--- | 3 | 81 | 33.96000 | 1.66E-25 |
| contig07470_1 | ---NA--- | hypothetical protein EHI_183570 | 1 | 26 | 32.89655 | 4.45E-09 |
| contig12244_1 | ---NA--- | ---NA--- | 1 | 25 | 31.62069 | 9.71E-09 |
| PpolyN1a08g07 | O08849 | Regulator of G-protein signaling 2, RGS2 | 1 | 22 | 27.82759 | 9.99E-08 |
| contig12659_1 | ---NA--- | ---NA--- | 1 | 22 | 27.82759 | 9.99E-08 |
| contig05590_1 | Q8H100 | GTPase-activating protein 8, AGD8 | 1 | 21 | 26.56897 | 2.17E-07 |
| contig12288_1 | ---NA--- | ---NA--- | 3 | 63 | 26.41714 | 1.91E-19 |
| contig12864_1 | ---NA--- | ---NA--- | 5 | 104 | 26.22337 | 4.67E-31 |
| PpolyN1a14d12 | Q07283 | Trichohyalin, TCHH | 1 | 20 | 25.29310 | 4.69E-07 |
| contig07949_1 | ---NA--- | ---NA--- | 1 | 20 | 25.29310 | 4.69E-07 |
